# Bodily expression of psychological distress in adolescents: a qualitative study

**DOI:** 10.1186/s13034-022-00476-9

**Published:** 2022-06-03

**Authors:** Maude Ludot-Grégoire, Aurélie Harf, Nour Ibrahim, Médérick Merlo, Christine Hassler, Joanne Rietsch, Charlotte de Bucy, Hervé Lefèvre, Jordan Sibeoni, Marie Rose Moro

**Affiliations:** 1 APHP, Hôpital Cochin, Maison de Solenn, 97 bv de Port Royal, 75014 Paris, France; 2 Université de Paris, PCPP, 71 av. Edouard Vaillant, 92100 Boulogne-Billancourt, France; 3 Université Paris-Saclay, UVSQ, Inserm, CESP, Team DevPsy, 16, Avenue Paul Vaillant Couturier, 94807 Villejuif, France; 4 Service d’endocrinologie, Hôpital Cochin, AP-HP, 27 rue du Faubourg Saint-Jacques, 75014 Paris, France; 5 French Clinical Research Group in Adolescent Medicine and Health (Groupe de Recherche clinique en Médecine et Santé de l’Adolescent), Hôpital Cochin, Maison de Solenn, 75014 Paris, France; 6 Centre hospitalier d’Argenteuil, Service Universitaire de Psychiatrie de l’Adolescent, Argenteuil, France; 7 ECSTRRA Team, UMR-1153, Inserm, Université de Paris, F-75010 Paris, France

**Keywords:** Bodily expression, Somatic symptom disorder, Adolescents, Qualitative research

## Abstract

**Introduction:**

The bodily expression of psychological disorders is one of the leading motives for consultations in adolescent medicine. The diagnostic entity corresponding to DSM-5 "Somatic symptom and related disorders" is sparsely used in the English-language literature, especially for adolescents. Qualitative studies on this topic mostly concern the experiences of healthcare professionals. This study seeks to explore the experience of adolescents expressing psychological distress through their body.

**Methods:**

This exploratory research took place in a Paris hospital department of adolescent medicine. Our sampling method was purposive. For inclusion, patients had to be aged 11–24 years, with a "somatic symptom disorder" meeting the DSM-5 criteria. Semi-directive interviews were proposed with visual narration inspired by photoelicitation. Thematic analysis allowed us to explore the data with an inductive approach.

**Results:**

Thirty adolescents were interviewed; they were 14–22 years old and mostly had somatic symptom or functional neurological disorders. Three principal themes emerged from our analysis of the interview contents: the personal, including bodily, experience of the disorder, the experience of relationships, and the question of what is visible through the body.

**Conclusion:**

This research allowed us to discuss the reversal of generations, the function of the DSM-5 diagnosis, illuminated by sociology, and finally, cultural pathways. It shows the importance of recognizing the reality of the adolescents' bodily feelings, reassuring them by ruling out serious causes, and supporting their search for meaning. It is important to think about a specific framework of family therapy that can make effective use of this experience of the reversal of generations.

**Supplementary Information:**

The online version contains supplementary material available at 10.1186/s13034-022-00476-9.

## Introduction

The bodily expression of psychological disorders is one of the leading motives for consultations in adolescent medicine [1, 2]. The nosographic approach is in near permanent mutation. It is currently defined in the Diagnostic and Statistical Manual, 5th ed. (DSM-5) as "somatic symptom and related disorders” (SSD) [3]. This diagnosis groups different clinical pictures together, specifically somatic symptom disorder, illness anxiety disorder, functional neurological disorder, psychological factors affecting other medical conditions, and factitious disorders.

All of these postulate that psychological distress is manifested by the body (or is the source of an exacerbation of a somatic complaint), whether or not an organic pathology has been diagnosed. SSD is defined as: *“one or more somatic symptoms … that are distressing or result in significant disruption of daily life … associated with excessive thoughts, feelings or behaviors related to these symptoms; although any one somatic symptom may not be continuously present, the state of being symptomatic is persistent (typically more than 6 months)."*

This diagnosis requires specifying if the "somatic symptoms predominantly involve pain", if they are persistent, and if the severity is "mild, moderate, or severe" [3]. The World Health Organization International Classification of Diseases, 11th ed. (WHO ICD-11) also offers a diagnosis that is essential a synonym of "somatic symptom disorder": "bodily distress disorder, unspecified" [4].

Although considerable SSD research takes place among adults, the term remains is rarely used in the general pediatric population. A recent systematic review of work in adolescents [5] showed that the term SSD is not often used in the English-language literature and that few specific studies of it concern this population. Although it is recommended by some authors [6, 7], others warn about the risk of overdiagnosis of a psychiatric pathology, especially when a somatic disease is also present [8, 9]. Among the explanatory factors, past trauma has been clearly identified, environmental aspects have been suggested (parental absence, difficult peer relationships), and neurobiological hypotheses proposed. Multidisciplinary management is recognized to be necessary [5].

Although several qualitative studies have been published on the subject, they mainly concern the experiences of healthcare professionals [10–12]. Only two qualitative studies have focused on adolescents: one from Switzerland [13, 14] of 10 adolescents with "medically unexplained symptoms," and the other Danish [15], of 11 adolescents with "functional disorders." These two studies, dating from 2015 and 2020, therefore do not use the diagnosis of "somatic symptom disorder", as defined in DSM-5 or ICD-11. Moreover, both studies present metathemes that flow from their analyses of the combined narratives of adolescents and their parents. Finally, the role of psychiatric care for these patients is sensitive: Hulgaard's study [15] underlines the difficulty of the moment of referral for a psychiatric consultation, which both adolescents and their parents experience as a tangible sign that the reality of the symptoms is not recognized. What meaning do adolescents attribute to these bodily expressions? Is there a place for psychological, psychiatric, and sociocultural theories in these adolescents' representations?

Psychosomatic theories (first developed in the United States) have struggled to find a place in Western medicine, between the medical and psychological models [16]. They rapidly leaned on key concepts of Freudian psychoanalysis (such as its economic foundation of drives—libido, cathexis, etc.) and mind/body dualism. These theories thus consider patients from the perspective not of their disease but rather of the identification in their psychic functioning of a somatization process that in turn depends on the quality of their mentalization.

Sirri and Fava (2013) [17] compared the diagnostic criteria in psychosomatic research (DCPR), developed in 1995 by an international group of investigators [18], with the then-new DSM-5. The DCPR comprises a set of 12 psychosomatic syndromes: eight concern the principal events of "abnormal illness behavior": somatization, hypochondriacal fears and beliefs, and illness denial. The other four syndromes (alexithymia, type A behavior, demoralization, and irritable mood) refer to the domain of psychological factors affecting medical conditions [17]. According to these authors, DSM-5 captures only a small portion of the information necessary for these clinical processes and neglects in particular important characteristics of psychological factors affecting medical conditions and abnormal behavior related to them.

In 2018, Van der Feltz-Cornelis et al. proposed a “specific research agenda” to identify “the main challenges and research priorities concerning SSD, bodily distress disorders (BDD), and functional disorders (FD) from a European perspective” [19]. Its high medical and societal costs cause SSD to “pose a substantial challenge to the population and health policy of Europe” [19]. They are “a burden for patients and their families” and “because of the high level of complexity at [the] diagnosis, treatment, health services and social level”, they form “a diagnostic and treatment challenge for general practitioners, occupational physicians, medical specialists, psychotherapists, psychiatrists and allied health professionals alike” [19]. Under these circumstances, we consider that patients themselves are the best experts to help professionals better understand their disorders.

This qualitative research aims to assess the meaning that these adolescents ascribe to these bodily expressions and the place they make for theories about them in their representations. We interviewed adolescents with SSD about their experience of somatic manifestations.

## Methods

This description of the methods we used follows the COREQ (Consolidated criteria for Reporting Qualitative research) guidelines (see Additional file 1).

### Setting

This exploratory research took place in a Paris hospital department of adolescent medicine. This multidisciplinary department manages distressed adolescents, whether their distress is somatic, psychiatric, or related to eating disorders. It includes an outpatient consultation clinic, a day hospital, and an inpatient unit. The study used noninterventional research, authorized by the Assistance Publique–Hôpitaux de Paris (AP-HP) ethics committee on June 15, 2020.^1^

### Sampling and participants

We used purposive sampling, that is, chose potential participants selectively and intentionally [20]. The inclusion criteria required that participants be adolescents aged 11–24 years, with SSD diagnosed according to DSM-5 [3]. This age range was chosen to include the full range of adolescent development and the societal understanding we now have of this life stage [21]. Moreover, we sought to vary the levels of disease severity (by including adolescents admitted for full-time hospitalization as well as outpatients), socioeconomic status, and culture of origin. The exclusion criteria were limited to adolescents in psychiatric decompensation, not considered able to handle a research interview. Two researchers, both psychiatrists for children and adolescents (ML and MM), conducted this qualitative study. The study was proposed to potential patients by their referring physicians, who were informed about the ongoing work conducted in the department about SSD and provided the adolescents with a written note explaining the study during a consultation.

We explained to the physicians, the adolescents, and their families that this study concerned the bodily expression of any kind of distress and that its objective was to obtain access to their experiences and representations. Participants were contacted by telephone. Patients (and when necessary, parents) provided written consent to the researchers after receiving full and complete information about the study organization, the research procedures, and its implications.

### Data collection

Semi-directive interviews of 60 to 90 min (see Appendix 1) were proposed to the adolescents, with visual mediation, inspired by photoelicitation and known to facilitate the emergence of oral narratives [22, 23]. Some 30 photographs were proposed at two points during the interview, all from the photobank (Photolangage®) of our department's psychomotor therapist (JR). From the 300 pictures she has, we set up two different photobanks (30 photographs each), chosen mainly based on the adolescent's gender, to facilitate identifications. The interviews were recorded on a dictaphone, then transcribed verbatim on a secure computer in the department, and immediately anonymized. Requests for clarification could be sent by email to the interviewees, after transcription, to clarify some unclear points or to ensure that our understanding of their point of view was accurate. All participants were asked to validate their transcripts. We continued inclusions until data saturation, that is, until the analysis of new data provided no new results [24].

### Analysis

We used thematic analysis to explore these data [25]. This method makes it possible to identify, analyze, and describe particular themes derived from the interview contents. Our thematic analysis used an inductive approach, that is, a process for coding the data without any reference to any theoretical concepts or preconceived ideas the researchers might have. This process is dynamic and iterative, and each new transcript led to the collection and subsequent analysis of additional data. The objective was to recognize and identify the similarities and differences between the analyses of each participant. The researchers were thus led not only to recognize recurrent patterns but also to integrate new issues as they emerged from the analysis. Table 1 summarizes the five stages of our thematic analysis. This phase was triangulated by a researcher who is a psychiatrist for children and adolescents as well as a family therapist (AH) and supervised by a researcher expert in qualitative methods (JS).

**Table 1:** Process of inductive thematic analysis

	Steps of the analysis	Rationale
Stage 1	Repeatedly read each transcript, as a whole	Obtain a global picture of the interview and become familiar with the subject's verbal style and vocabulary Each new reading of the transcript might also provide new perspectives
Stage 2	Code the transcript by making notes corresponding to the fundamental units of meaning	Make descriptive notes using the participants’ own words Pay particular attention to linguistic details, such as the use of metaphor
Stage 3	Make conceptual notes through processes of condensation, abstraction, and comparison of the initial notes	Categorize the initial notes and reach a higher level of abstraction
Stage 4	Identify initial themes. Provide excerpts from the transcript that illustrate the main ideas of each theme	Themes are labels that summarize the essence of a number of related conceptual notes. They are used to capture the experience of the phenomenon under study
Stage 5	Identify recurrent themes across transcripts and produce a coherent ordered table of the themes and subthemes	Move from the particular to the shared across multiple experiences. Recurrent themes reflect a shared understanding of the phenomena among all participants. During this more analytic stage, researchers try to make sense of the associations between the themes found

## Results

We interviewed 30 adolescents with some form of SSD, aged 14–22 years, mainly female (25/30), and across the entire socioeconomic spectrum. The largest group had an SSD in the strict sense of the term (14/30). The others had related disorders: functional neurological disorders (9/30), illness anxiety disorders (3/20), psychological factors affecting other medical conditions (3/30), and finally one with a suspected factitious disorder (1/30). Table 3 in Appendix 2 summarizes their characteristics.

Three principal themes emerged from the analysis of the contents of these interviews: the bodily experience of the disorder; the disorder’s effect on their relationships; and the question of what is visible through the body. These themes illuminate the experience of adolescents expressing distress through their bodies. We present them here, illustrated by significant excerpts from the transcripts and several illustrative images. Table 2 summarizes the main themes.

**Table 2:** Main Themes

1. The personal experience of the disorder	a. Bodily experience b. Quest for meaning c. Death anxiety
2. The disorder's effect on their relationships	a. Family reactions *Disbelief, abuse* *State of dependence* *Family history* b. Reactions of peers and school c. Medical care providers' reactions
3. The question of what's visible through the body	a. What the adolescent lets be seen b. What others can see c. What Medicine does not see *Sign of the disease's nonexistence for physicians* *And in this nothingness, the psychological cause finds its place* *The families questioned the limitations of medicine*

### The personal experience of the disorder

#### Bodily experience

**Fig. 1:**
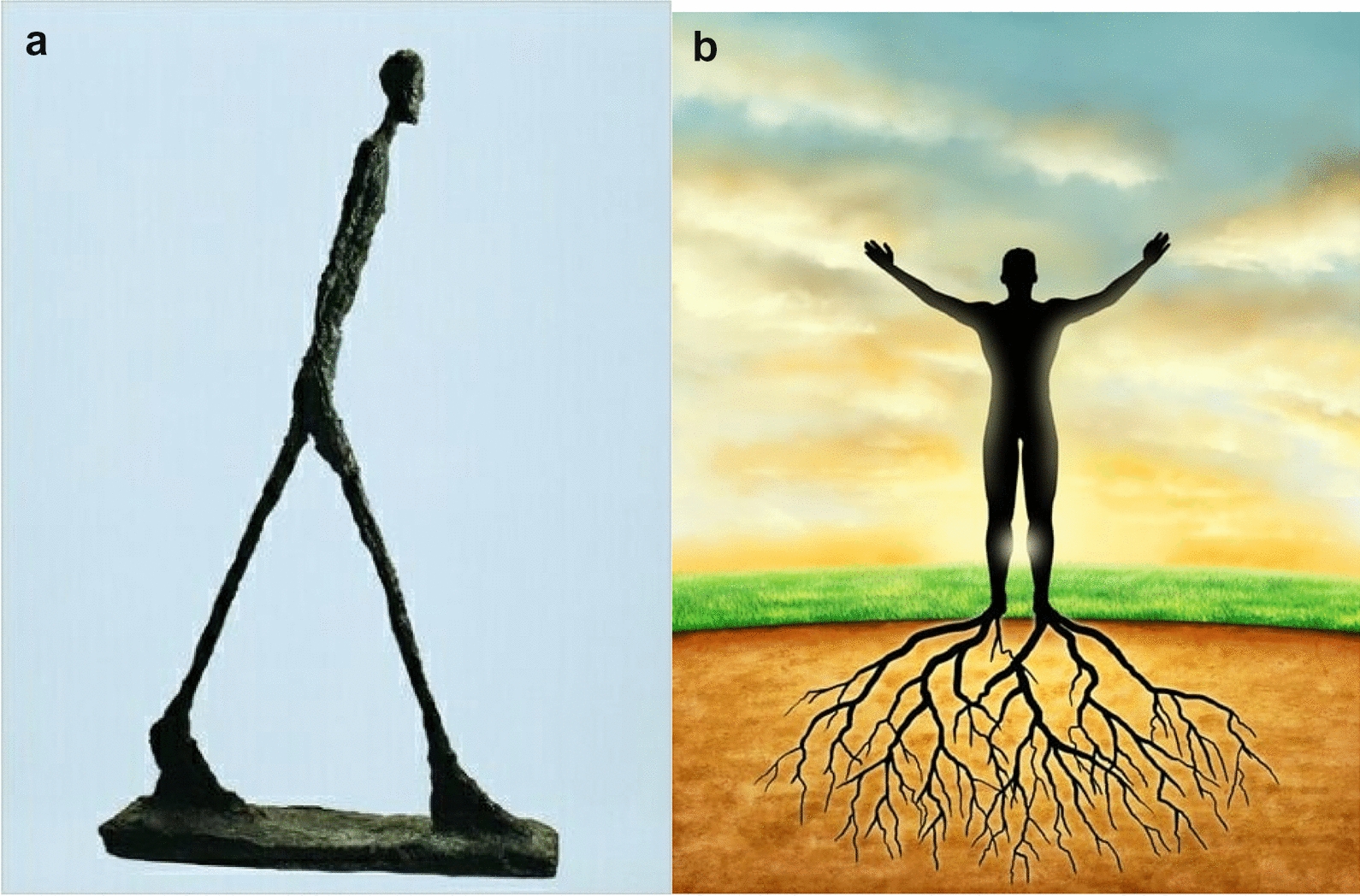
**a**, **b** Pictures associated with blockage

Most adolescents (18/30) reported symptoms of pain, most frequently joint pain (12/18); next came neurological symptoms (10/30), and finally symptoms of (or more or less associated with) bodily oppression (4/20). Because of these symptoms, especially when they affected joints, adolescents described an experience of motor limitation, sometimes even "blockage" (see Fig. 1a, b, images chosen by 8/30 patients). Accordingly, Emma had the impression "of having legs that were worn out … a little rusty sometimes." She added, "I'm not a grandmother but I imagine that grandmothers feel that." Donovan also expressed his feeling of "legs [that are] giving out" and added that "I fall pretty often, because I have the impression that my legs fell asleep for a fraction of a second." Valentin described an "impression that my body is eating me, nibbling at my muscles," and continued, "I have the feeling that whatever I do, the pain gets worse, that I'm wasting away." Laurene linked her motor limitation to the slowing of time, from the image below: "weight in my legs … I imagine it's going to get worse, maybe more slowly."

**Fig. 2:**
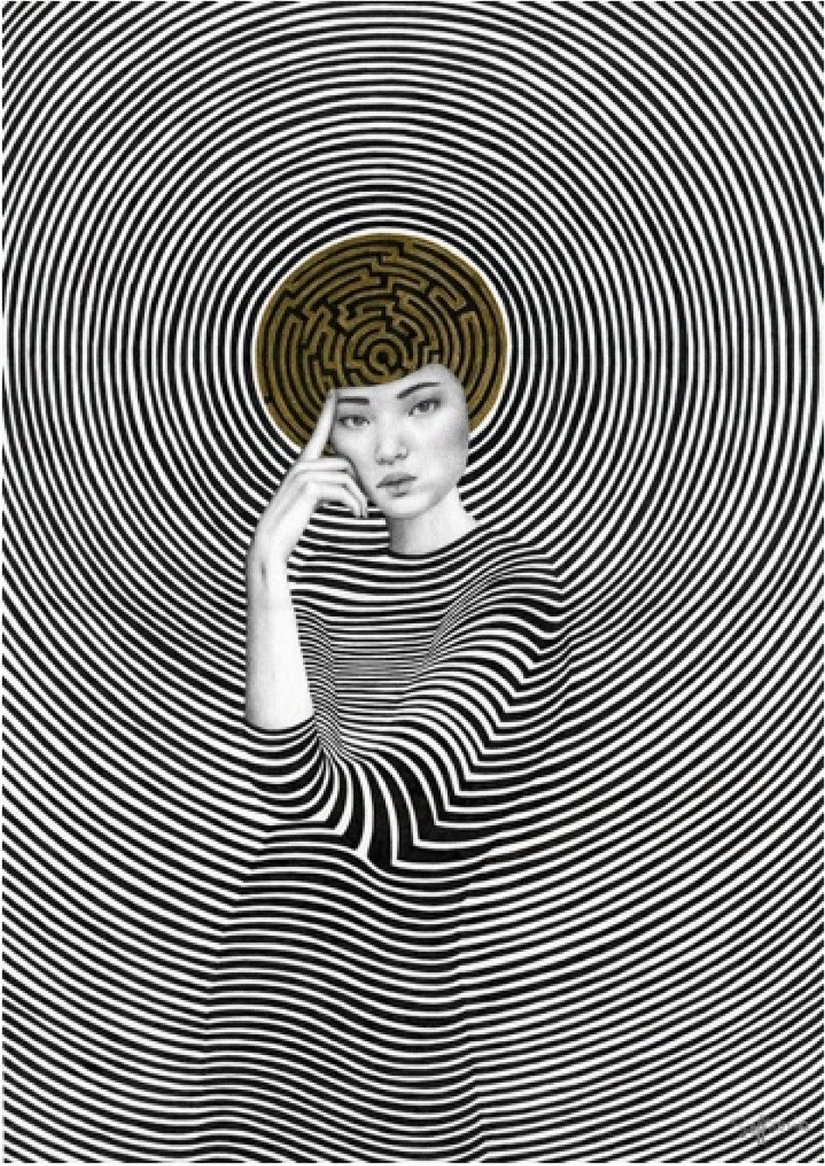
A pensive woman

From the cognitive perspective, also, Lucie chose (for her visual narration) the image below of a pensive woman (see Fig. 2):

##### …the fact that she has her hand on her head, it's as if she's thinking about what she could say or trying to find the right word, because that happens to me a lot, to have to find the right words.

Other adolescents talked about their difficulties in concentrating because of these somatic manifestations. Maxime identified "two aspects of this stomachache, which are that sometimes it's so bad that it's hard to walk, but sometimes even when it's not as bad, it's hard to concentrate."

#### Quest for meaning (Fig. 3a, b)

**Fig. 3:**
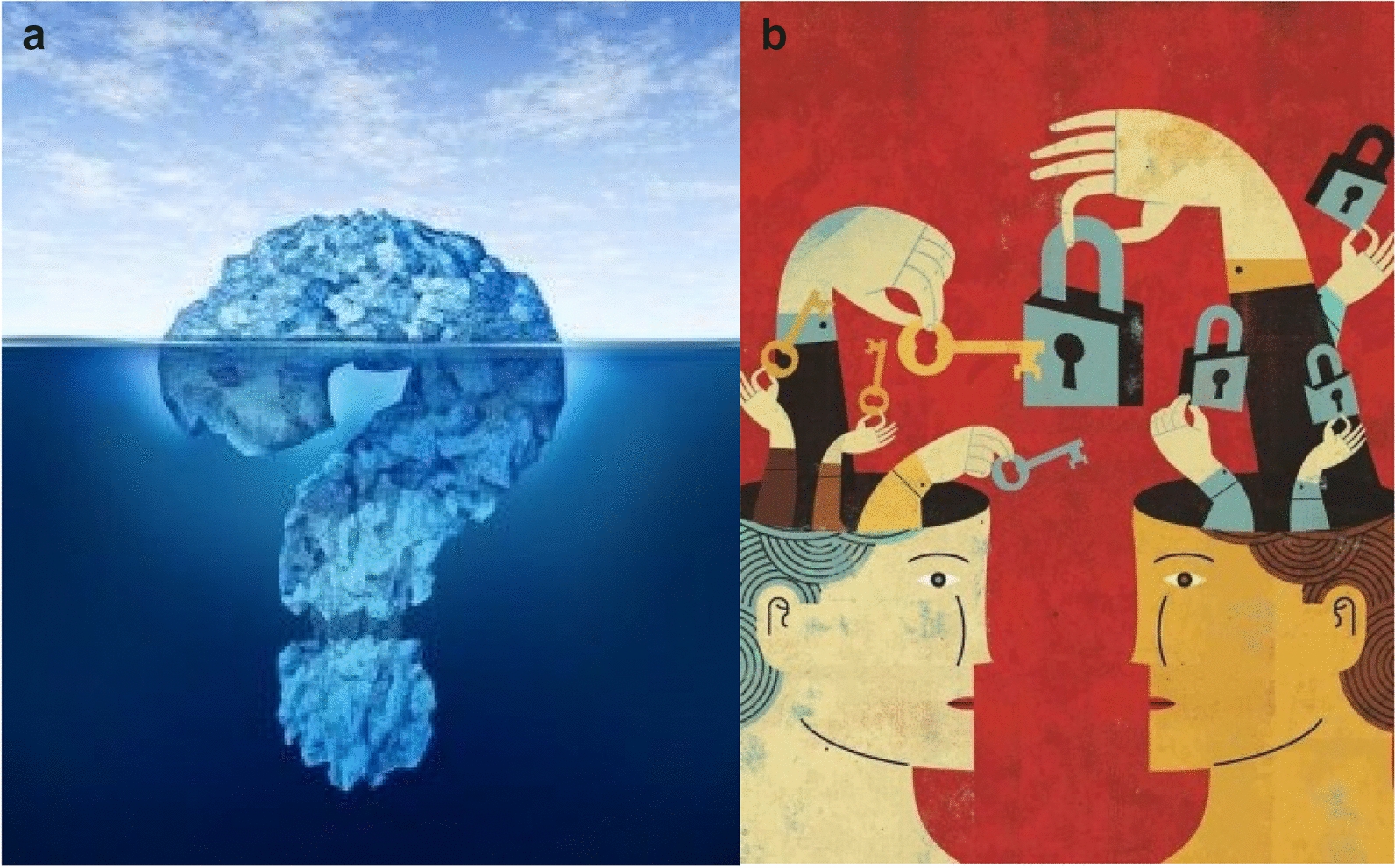
a, b Quest for meaning

**Fig. 4:**
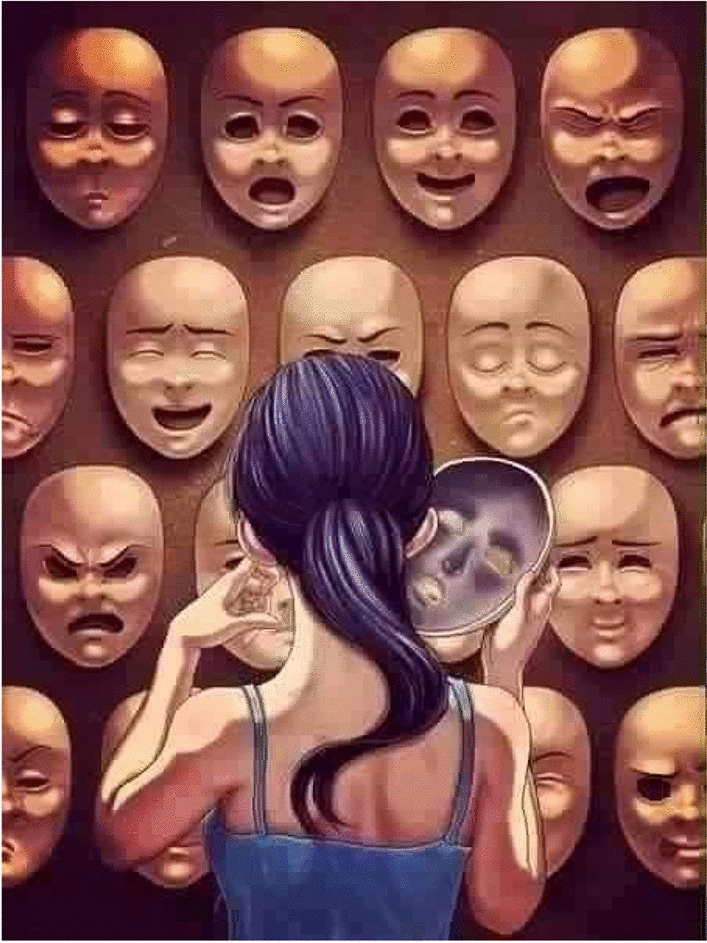
Range of emotions

Some adolescents hypothesized that these somatic manifestations are an expression of how badly they feel — of their ill-being. For Amina, it could be "a sort of release, but one that hurts a lot and is very uncomfortable"; she added, "I'm freeing myself perhaps from a weight that I've been carrying for years." Sophie's image of the pain in her sternum was more detailed: "it's as if someone wanted to leave but…it's like someone is pushing to open a door but stumbles." This image in turn resembles that of Jade, who said, "it's as if something's inside and you couldn't manage to channel it and as a result there was something accelerating to get it out." Maryam, who described herself as "not expressive," identified the emotions that could trigger somatic manifestations: "when I'm going to laugh, I'll fall, when someone tells me sad news, I'll fall." Similarly, Maxime recognizes "problems in identifying and expressing my emotions," perhaps the source of his pain; a work-up for autism is underway. Esther also had questions about the management of her emotions, expressed through the image she chose (Fig. 4):

It makes me think of emotions, in fact, I don't know which to choose when I'm having a [fit]. Because in fact when I have a [fit], I really have no emotions, my face, my body is blocked; it’s as if I were paralyzed."

Later she asked questions about "the accumulation of emotions" at the origin of these fits. Laurene was the only one to talk about "my body's particular reactions to stress," focusing on the stomach, "the most fragile organ, the weakest."

Without explicitly mentioning a traumatic cause, some adolescents associated their pain chronologically with a potentially traumatic event. Thus, Diane noted that "these [episodes] had started in a still more disabling form, it was a little after my grandmother's death … it's true that the dates coincided." Samira pointed out that "these anxieties really developed after my heart transplant." Marthe, who has unspecified low back pain, reported a traumatic incident several years earlier: "in middle school, I fell on my head … but they only did a CT scan of my head, although I also landed on my back." Esther's first psychogenic non-epileptic episode followed a liver biopsy and was followed in turn by anxiety attacks: "they took a piece of my liver, and … my body, it reacted." As for Yasmine, who had a long health care pathway, she recently realized she had been a "victim of incest": "maybe that's the reason I have anxiety attacks, and maybe also why I'm paralyzed." Finally, Marie-Claire associated her fainting spells with a "traumatic cause" that she did not discuss further.

Hawa, whose mother's family came from Côte d'Ivoire, mentioned their cultural etiological theories^2^ and especially the possible supernatural intervention: "did some evil spirit enter my body?" For Amina, whose parents came from Guinea, the loss of control of her body often gave her the impression of "fighting against phantoms, people, voices;" she added, "I don't even know how to explain that, I can't. If it was spirits or ghosts or I don't know what." Maryam's family, originally from Morocco, also suggested a role for the "evil eye" in her non-epileptic psychogenic attacks triggered during intensive athletic training sessions and conflicts with her trainer: "I said to myself that it's possible." Yasmine, whose parents came from Algeria, had similarly thought of witchcraft:

I didn't believe it until someone insisted and we went to see someone else who knows how to deal with it and who found a sign of witchcraft, as they say, with writings, hair that is mine, a piece of clothing, rubber bands, and a photo of me. Then I said to myself "wow."

Many adolescents described their bodily sensations with words related to combat, to fighting. Others, finally, seems to search less and talk more of fate. Aurelie, for example, said "it could have happened to someone else but it happened to me." Similarly, Myrtille, despite having a genetic pathway to understand her polyarthritis, thinks that "it's also fate, in fact." Marie let herself be convinced of the anxiety hypothesis: "everyone told me that, I ended up by telling myself that too." But for Andrea, stress constitutes "the emerging face of the iceberg."

#### Death anxiety

The question of a serious underlying disease thus arose for some adolescents, who expressed fears of death. Mehdi, for example, admitted, "given that I am really afraid of dying, well, inevitably, I get anxious, necessarily there's stress and anxiety." Often, these anxieties are shared with other family members, such as Lucie with her mother, who had imagined the worst: "It was so violent that when I was little, I thought, even my mother thought, I had a brain tumor or something like that." Andrea too shared these fears of death: "I kept seeing myself die … I was wasting away, and I expected to die." She said that her parents did too: "It was also very hard for them because obviously when your child is dying and then all the doctors say, ah, it's stress, don't worry!'".

Thus, the personal experience of the disorder is expressed in the adolescents' discourse through their bodily experience, which, especially when it affects the joints, "blocks" them and summons representations that belong to an aging body. The quest for meaning is triggered at the individual and intergenerational levels, and the symptoms can engender substantial anxiety about death.

### The disorder's effect on their relationships

#### Family reactions

##### Disbelief, abuse

The adolescents recounted the doubts they heard in the discourse of their parents and siblings about the authenticity of their complaints. Thus, Justine mentioned the reaction of her mother, who one day threw out her knee brace: "she called me a liar and said she doesn't believe it, she says it's an act." Similarly, Mehdi described the comments of his 16-year-old sister, who regularly rebuked him: "you're going too far." Marthe felt her family suspected she was using the pain "as an excuse for special treatment." Jade detailed her brother's reactions to her pain: "he always calls it into question, by minimizing my suffering … or insinuating that the suffering is not real or legitimate and that it's all an act or something." For Laurene, "there was a moment when my parents stopped believing me; they thought I was deliberately having stomachaches to not have to go to school or leave the house."

##### State of dependence

These symptoms, which limited the adolescents' motor and sometimes cognitive capacities, engendered dependence on those around them. Samira described her dependence on her parents: "I can't go out anymore without one of my parents." Hawa expressed her dependence more specifically on her mother: "my mother, because she works at home, she could help me, anyway, when I wanted to eat, especially because I couldn't carry my plate, so she helped me." Because of her disabling hip pain, Marie reported that she often called her grandparents, with whom she was living, even at night "to take me to the toilet, bring me something to eat in bed." Yasmine recounted her longest episode: "Two months like that, with only my mother to carry me, take me to the bathroom, help me shower; it was unbearable."

##### Family history

Some adolescents recounted similar symptoms in their elders. Maxime reported his mother had the same abdominal pain, which had started at the same age and continued to this day. "She didn't have a diagnosis of a physical disease, so it was a little complicated for her." Marine referred, without associating it to what was happening to her (algodystrophy requiring the use of a wheelchair and confinement to bed), to her paternal grandmother, who died of a stroke but had spent several years in a wheelchair or in bed all day. Yasmine talked about her grandparents' symptoms; "one had trouble walking, something to do with the ankle;" the other was "totally paralyzed by a stroke." On the subject of her low back pain, Marthe told us that "my mother's sister, she had an operation on her back, on her vertebral column at around my age … and …still has back pain."

Other adolescents reported serious diseases sometimes diagnosed with what they perceived as excessive delay. Thus, Justine said of her father: "he had a disease that was just discovered and that no one believed he had … because the doctors didn't find anything … since he was young, he looked, he had vertigo, and, well he had a stroke." Hawa described a fatal diagnostic delay for her paternal grandmother, who.

… always had problems of constipation and all that … so we stopped asking questions, and then we discovered she had cancer 3 months before she died. So it was very fast, we didn't have time to understand she had cancer until … it was already finished.

Finally, Valentin spoke about his maternal grandfather, who had a "throat cancer that he hid" and that he died of; and, two months later, of his godfather's death "on the operating table, of a disease that's normally benign."

Accordingly, their families were strongly present in the adolescents' reflections about the place of pain and illness over several generations.

#### Reactions of peers and school

Peers asked many questions, more or less aggressively, about the meaning of the symptoms expressed, especially when they fluctuated a great deal. The adolescents felt pressured to justify themselves. Leslie reported having "had lots of insults about the disease because one day I had crutches, and another day, I didn't." Myrtille disclosed how her peers mocked her for her fluctuating symptoms: "I would arrive in a wheelchair, the next day I didn't have it, then the day after, I was on crutches, and then after that …nothing….they treated me as a liar …' you make up a disease to be noticed'."

The adolescents perceived a climate of suspicion around them, whether by other students, their teachers, or staff. It was more or less directly implied that they were feigning their illness to justify absences or obtain particularly favorable treatment within the establishment. Justine thus reported that "the others say I do it on purpose, to not have to have gym class," while Aurelie complained that "the staff doesn't want me to go upstairs before the others … because they think I'm faking, some of them anyway."

#### Medical care providers' reactions

In the absence of a medical diagnosis, they described their reception (by healthcare professionals, especially in emergencies) as difficult, sometimes even perceived it as rejection. Hawa told us about her experience of an emergency room visit: "they said to me, 'oh, well why are you here?' … though if I'd had this or that disease, I could have come, they'd never have asked me that question." Diane went to the ER recurrently for bouts of pain, accompanied by her mother. "Each time we went to the hospital they wondered if I wasn't exaggerating a little." Marie-Lou reported the reactions of hospital staff and her perception of the quantification required on the pain scale.

It's hard to be taken seriously. I know that they ask me all the time for this scale. And I always perceive the pain at 7–8 … But when I say that to the doctors who ask me or even to the nurses, I see their reactions: they have the impression…not that I'm lying but that my perception is too high compared with the scale or something like that.

Andrea spoke of her situation of "medical mistreatment” because of the words used: "'you do that to get attention, you're lying, stop making such a fuss. There are people who are really suffering, there are people who have cancers.'" For Marine, who has algodystrophy, "[it] was very complicated with especially the doctors, because they said openly at the hospital that I was a liar, so that marked me." For Nassim's rheumatologist, "it wasn't a real pain at the joints, but for her, it was more that mentally I had a problem."

Because of the absence of a medical diagnosis, the adolescents reported very strong reactions and difficulties in having their distress heard and recognized by their families, in continuing school in good conditions, and in receiving care from health professionals.

### The question of what's visible through the body

#### What the adolescent lets be seen

**Fig. 5:**
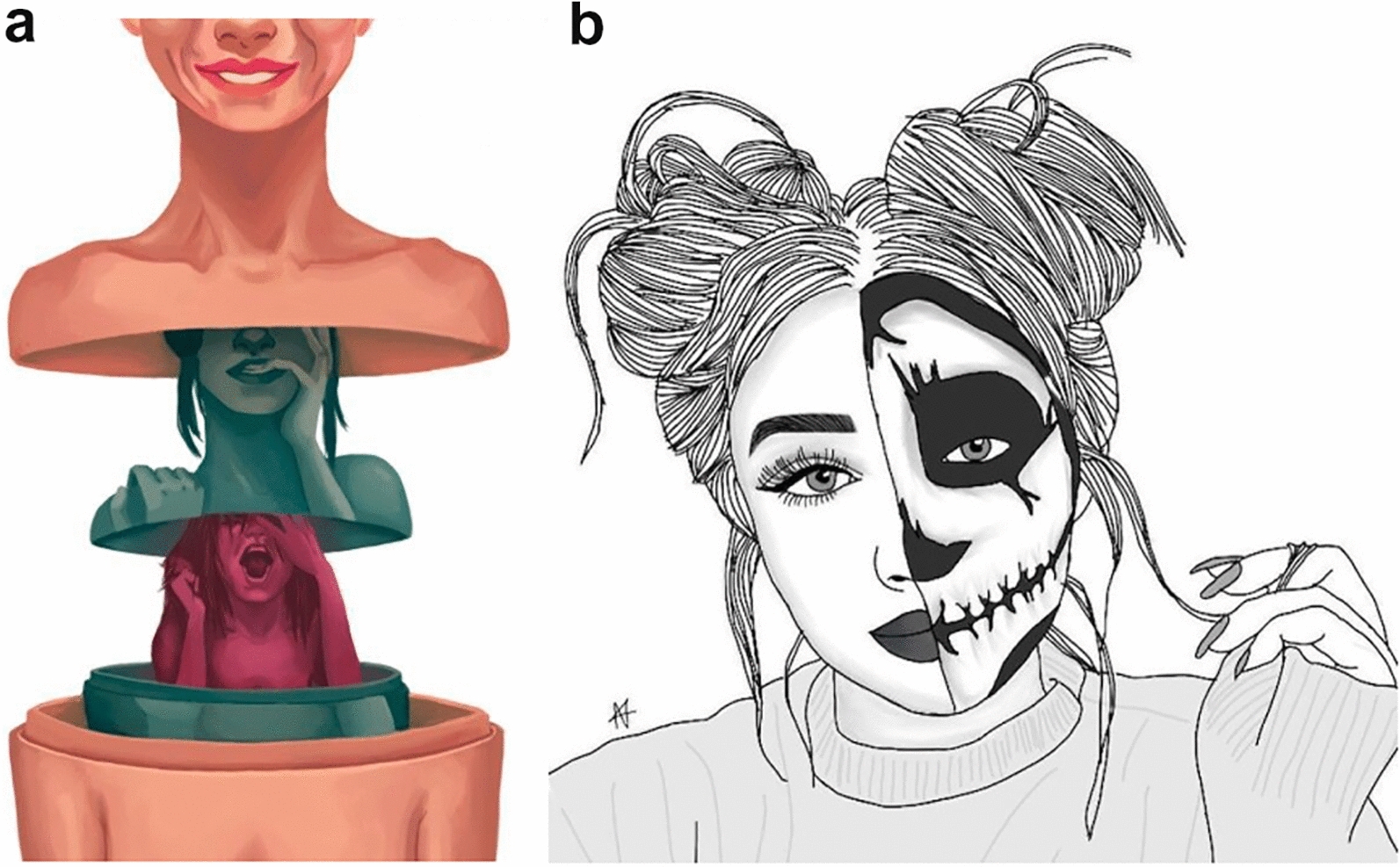
**a**, **b** Russian dolls and the double face.

Seventeen of the 30 adolescents said that they forced themselves not to show too much of their distress to others. Thus, Emma tried "to put a good face on, let's say, to not worry the family too much … I've never liked having too much attention." Leslie said she "tries her best to make [her] disease as invisible as possible to be able to have some time without thinking about it." Pauline calls herself basically "a person who smiles really a lot …but from year to year it gets worse and worse, but I kept smiling, I made believe … so I see it a little as armor, or a costume." Donovan said about his migraines that they are "not perceptible to others" and that he "tends not to want to make them visible, but to hide them more than anything else." Finally Marthe forces herself to "to always show the same emotion, that of joy, to be the person who's always smiling, in a good mood so that people see that I'm well." Marine chose an image of Russian dolls (stacked each within another) (see Fig. 5a):

…where you hide your emotions, you hide what you're feeling, what's not going well, it's exactly what I do every day. Because I don't want people to worry or my parents to have more to worry about than they already have; and I am naturally very private, I need to have my little garden.

For some adolescents, this can be explained as a mark of modesty. As Leslie said, "I want this to be private, I want it to be for me … it's my personal life." It can also be a way of protecting the others, as for Amina, "I smile because I've always smiled; these people don't need to see my pain or to have it either; it belongs to me." Ten adolescents thus chose the image of the Russian dolls or the double face (see Fig. 5a, b).

#### What others can see

The adolescents talked about how it was impossible for others to understand their symptoms, because of the "interior nature" of their distress, that others could not represent it to themselves because it was not visible from the outside. Accordingly, Sophie affirmed that "… it's interior, no one sees it [the pain] … You'd have to be in my body to understand what I feel." Hawa said it too, in her way:

… on the outside, you can seem all beautiful and rosy, after all, we don't see it; since it's a psychological problem, it's not a problem. When we break a leg, we see that there is a cast; this, you don't see, it's inside.

For Marthe, when others look at her, "I have the impression that it causes a reflection and it hurts me, from the pain." Accordingly, five respondents chose the picture of a skeleton, an image that calls into question the borders between interior and exterior and the effects of the exterior gaze.

#### What medicine does not see…

##### Sign of the disease's nonexistence for physicians

The word "nothing" was used regularly to describe the absence of objective signs on clinical, laboratory, or imaging examinations. Amina recounted her experience with physicians this way: "I have doctors who told me over and over, 'nah, you have nothing, you have nothing at all …we see nothing!' Who mostly stopped at medical examinations." Mehdi also reported his experience: "I've also had blood tests that detected nothing in particular; I had an allergy test for respiratory allergies: I had nothing." For Lucie:

…at one point, they kept me for a week, they did everything, MRI, ophthalmology, they did all the ORL (ear, nose, and throat) things, hearing, etc.… I did everything, I did an electroencephalogram to see if it wasn't epilepsy…and at the end, they told me, 'Lucie, there's nothing.'

##### And in this nothingness, the psychological cause finds its place

This is how Diane explains how the psychological diagnosis was formulated: "I think that it was at a hospital with a neurologist who I saw often … And it's the term 'somatoform' that she used when she saw that there was nothing neurological." For Leslie, because she didn't have any "rheumatoid factor", "my rheumatologist told me that it was psychological." After "having a brain CT scan, seeing an ORL, an ophthalmologist, and seeing a physical therapist for sessions to dissolve the crystals in her ears," Pauline finally concluded that "we understood that it was truly psychological: there was nothing." For Marie, who was observed for a week in a pediatric department for these non-epileptic psychogenic fits, "finally they found nothing, they said I was experiencing burnout." For Manon, depression appeared to be the only remaining possibility:

Normally, you take tests to see if a person is depressed, and if they score more than 10 out of 20, they're considered to be depressed, but I had 3. But they said to me, well since you don't have anything else, you have to be depressed.

##### The families questioned the limitations of medicine

In this medical void and with a psychological cause now the hypothesis, the adolescents and their families interrogated the limitations of medicine.

Lucie reported her reaction, "they told us, well, listen, there's nothing. So we said, well, medicine can't do anything, so we'll look for alternative medicine and I started to do acupuncture, which helped me a lot."

Leslie contacted a patient group for nonspecific joint pain:

…well since she [the rheumatologist] couldn't figure out what it was, she told us there was no point in continuing to see her, that we could leave. So we spent 6 months without a doctor then …with the disease we…there's a patient group for that, the "Courir" association that works for this type of person.

Emma said, about rheumatoid purpura, which she suspects she has: "it's been pretty complicated for this type of disease, not very developed or considered as a possibility in France." Hawa, who suspects she has chronic Lyme disease, expressed her doubts:

Isn't there a disease underlying all my joint pain, especially as there was a trace of Lyme disease in the blood samples: is it that or not? Because Lyme disease is a very vicious disease … In the United States there are a lot of specialists in it, and patients are treated well, but in France there are lots of physicians who don't even consider chronic Lyme disease, and that's a big problem.

In the absence of objective signs recognizable by Western medicine, the adolescents and their families continued their search, even when psychological distress has already been suggested.

We note in this thematic analysis the existence of two negative cases, one adolescent who is different from her peers and from the medical world: Myrtille, who has juvenile rheumatoid polyarthritis, meets the criteria of DSM-5 because stressful events aggravate her symptoms. The underlying disease was recognized here and specifically named, and this young woman underlined the support she has received from medical professionals. The disease is described here as making her vulnerable to the exterior world and her peers in particular, and she described multiple traumatic events. Myrtille also expressed her desire to be like everyone else and especially not to stand out from other adolescents. The other negative case is Sarah's, very probably a factitious disorder, although her parents have endowed her with several diagnoses (Ehler-Danlos and Lyme); her experience does not at all resemble that of the other adolescents, her description of her symptoms is very sparse, and her narrative extremely vague.

## Discussion

These 30 adolescents provided a rich and detailed description of their experience with SSD. The principal themes that emerged were: the personal experience of the disorder; its effects on their relationships with family, peers, and care providers; and the question of what is visible through the body. All these themes resonate with the process of adolescence: the changing body escapes the teen's control, because of hormonal but also neurodevelopmental changes and modifies how he or she deals with emotions or with social situations [26]. The relational experience of the disorder takes its place within the adolescent process of separation-individuation described by P. Blos [27]. In these adolescents with weakened bodies, dependence on adults is massive and impedes adolescent autonomy. The question of what is visible through the body echoes the importance of secrecy in adolescence, to guarantee the autonomy necessary for functional thought [28]. Thus, these adolescents spoke about intimate bodily sensations, which are difficult to share, while showing crutches, wheelchairs, etc. and saying that they suffer from the non-recognition and invisible nature of their difficulties. From the psychosocial perspective, the psychodynamic aspect of bodily sensation is important: recent work shows that emotions coming from social interactions correspond to the activation or deactivation of specific body areas [29].

These themes raised the issues we discuss below: the reversal of generations; the function of the SSD diagnosis, illuminated by sociological studies; and finally the issue of culture.

### Reversal of generations

The adolescent body, which incarnates the heritage of the parents and is "the site where identification is expressed through resemblance to family members" [30], becomes particularly worrisome. These family representations that are too similar or else too frightening appear to dumbfound this identification process. But what appears more notable and original here is the disruption or reversal of the generational order that appears in the adolescents' experience. They express bodily feelings that belong to an aging body. On this subject, we find in Jones's "Phantasies of the reversal of generations" [31] the proposition that any child may fantasize becoming the parent of his or her parents, either by desire to take revenge against the parent or by identification with a grandparent. Guyotat described the "delusions of filiation" that underpin a loss of the temporal limits of the ego, "so that traumatic events experienced in the previous generation would pass as an inclusion in the psychic functioning of the subject, favoring errors, confusion of dates, projections, etc." [32]. The bond of filiation thus represents "a true device of genealogical transmission." Grammatical tenses are accordingly confused in the interviews, with a mixture of past, present, and conditional tenses. In his work on unconscious psychic transmission, Ciccone [33] invokes A. Eiguer's "representation of the intergenerational object" [34]. This notion emerges from observations of correspondence between some patients' symptomatic expression and fantasies attached by them or their families to ancestors. They are an unconscious fantasmic reconstruction of sometimes traumatic events to which all family members adhere [33, 34].

Might there be a particularly important invisible family loyalty among these adolescents? It could be invisible loyalty in the sense intended by the US psychiatrist, Boszormenyi-Nagy in his contextual therapeutic approach to unconscious or indirect family loyalty [35], beyond one's parents and for several generations. A specific theoretical framework for a family-based approach thus appears necessary to involve the grandparents in particular in understanding the adolescent's bodily manifestations.

### The function of the SSD diagnosis, illuminated by sociological studies

The adolescents spoke quite a lot about their difficulties in naming their symptoms and their distress that they could not. In a recent literature review of this subject [4], only 10 of 82 included articles used the term SSD in their titles. What is the function of this diagnosis if it is not used? According to health sociologists, the function of a diagnosis is to validate what is considered a disease, offer explanations, and give coherence to the patients' symptoms; legitimate the disease, thus enabling patients/people with symptoms to accede to the role of patient; provide them a means of access to care and facilitate their management; and serve as the foundation of medical authority [36]. It is a "starting point, the foundation from which the meaning and experience are developed [37]." It is as if we did this study in the opposite direction by describing the meaning and experience of adolescents who have no diagnosis. The adolescents interviewed in this study feel that society is denying their bodily feelings: they experience a disorder, the reality of which is challenged by the medical profession, their family, and their friends. This can be discussed through the prism of the social sciences. Do we overmedicalize a discourse of pain in order to give it an acceptable form? Or are these disorders the expression of changes in society itself? The philosopher Ian Hacking talks of some disorders that emerge transiently in particular conditions, which he names ecological niches [38].

What the adolescents are actually questioning is many of the limitations of medicine, doctors' ability to rule out an organic pathology in view of the patients' sometimes dramatic family history, and their availability to hear the youths' distress and provide them with empathy and guidance in the process of adolescence. Doctors' power and knowledge are challenged here, and their powerlessness to make a diagnosis is pointed out. The adolescents are not recognized in their distress but the doctors too are invalidated in their very identity.

### Cultural issues

In our population, more than half of these adolescents have migrant origins: one young woman is a first-generation migrant, and 17 are children of migrants. Among these adolescents, some have presented cultural hypotheses in questioning especially potential interventions of the invisible world. Socio-cultural theories accentuate the meaning rather than the cause given to the senseless entity that is disease [39]. The cause in these theories belongs to the technicians and specialists, but does not exhaust the meanings given to it by those who live this event [40]. Thus, in all cultures, patients are going to find meanings in cultural etiological theories, theories that are moving and never fixed, *“reworked by all the events and multiple contributions of a given context specific to a time and place; they are transmitted and transformed from one generation to another and are universal in the sense that each culture promotes their emergence *[40]*.”* This involves, as Zempléni says, being able to "make sense of what is senseless [41]." In this regard, we believe that further studies should investigate how these adolescents with migrant origins cope with somatic symptoms.

The last meta-theme, that of the question of what is visible through the body, derived from the experience of 30 adolescents, appears to us to require development in a subsequent article, because of the rich transcultural underpinnings to be discussed. Thus, the management of emotions, ways of being cultural, cultural defense mechanisms in the sense intended by Devereux, that is, preestablished common methods likely to attenuate anxiety or stress [42], as well as questions about the secrets and initiation into the quest for meaning conducted by the adolescent, and finally the limitations of Western medicine are all aspects of this study that should be developed in subsequent specific analyses. Supplementary interviews with the migrant population may then be necessary, with a secondary analysis of the participants' data. It would be appropriate in this case to question as well first-generation migrant adolescents, with an interpreter available. Moreover, interviewing the parents of the adolescents in this study would probably provide us more access to etiological theories and therapeutic logics.

### Strengths and limitations

Our study is one of the very few qualitative investigations reporting the perspectives of adolescents with SSD, and to the best of our knowledge, it is the first to present narratives from 30 adolescents and young adults with SSD. Furthermore, we used visual mediation to facilitate the emergence of oral narratives. The principal limitation of our research concerns the broad age group eligible for inclusion. Nonetheless, only two adolescents were younger than 14 years, and their narratives did not differ. There was one negative case (apart from Sarah, previously mentioned): Myrtille, 18 years old. Her narrative stood out from the rest of the interviews, probably because she had an underlying condition (rheumatoid arthritis). Interestingly, in criticizing this diagnosis, Frances asks clinicians "to ignore this SSD diagnosis" when an organic pathology is diagnosed. She points out that if a psychiatric diagnosis must be formulated for people worried about their medical problems, the most benign would be "adjustment disorders [8]."

## Conclusion

This exploratory qualitative study points out the importance of recognizing the reality of adolescents' bodily feelings and pain and promoting their verbalization. Reassurance that a serious cause has been or is being ruled out is essential, in view of the fear of death experienced by adolescents and sometimes their families. The search for meaning must be supported at the individual level, while taking into account the adolescent's privacy and quest for meaning, and at the family level. It is also essential to consider the portion of the adolescent’s quest for meaning that remains unknown. Management of these adolescents requires excellent coordination between healthcare professionals and even a form of synchronization between somaticians and psychiatrists, so that care by some never cancels out that of the others. Finally, our findings have implications regarding family therapy. It appears important to think out a specific framework of family therapy taking into account the adolescent's experience of the reversal of generations: it may in particular involve grandparents to enable the development of the things left unsaid between the generations and to reposition adolescents to a place that diminishes their guilt and allows them to overcome their conflicts of loyalty. Further studies will investigate subjective experiences of parents (to better approach cultural etiological theories) and how adolescents with migrant origins cope with somatic symptoms.

### Supplementary Information

**Additional file 1.** COREQ (COnsolidated criteria for REporting Qualitative research) Checklist

## Data Availability

The raw data supporting the conclusions of this article will be made available by the authors, without undue reservation.

## Appendices

### Appendix 1

#### Interview guide for the adolescents

Dear Adolescents, Your doctor has suggested that you participate in this study which concerns the bodily expression of any kind of distress.

1. I suggest that we agree about what we are going to talk about: what do you call what happens/has happened to you? [We will use this term this term throughout the interview]
2. I suggest that you pick out three photographs from this set that best represent what's happening to you. Why did you choose these photos?
3. How does it manifest?
4. Can you tell me about a lasting or significant memory?
5. Why do you think this happened?
6. What worries you most about it?
7. How have the doctors talked to you about it?
8. How has your family reacted? and your friends?
9. What has this changed for you?
10. How do you think that it's going to develop?
11. What has helped you and what have you tried that did not help you?
12. Here is another series of photographs. Could you choose the one that most resembles you?

### Appendix 2

See Table 3

**Table 3:** Participants’ characteristics

First name*	Age	Repeated a year or last diploma obtained	Currently attending or not attending school	Family situation	Relation to migrants	Parents' socio-professional category or educational level	DSM-5 diagnostic subcategory
Diane	16	Middle-school diploma	In school	Lives with parents	Child of migrants	Mother: manager Father: manager	Functional neurological disorder
Sophie	22	Vocational Baccalaureate*	Post-secondary studies	Lives with parents	0	Mother: intermediate occupation Father: manager	Illness anxiety disorder
Amina	22	French Baccalaureate	Post-secondary studies	Lives with parents	Child of migrants	Father: employee Mother: employee	Functional neurological disorder
Justine	17	Middle school diploma	In school	Lives with parents	0	Father: employee Mother: employee	Somatic symptom disorder
Laurene	16	Middle school diploma	Disconnected from school	Lives with parents	Child of migrants	Father: Intermediate occupation Mother: Intermediate occupation	Somatic symptom disorder
Emma	17	Middle school diploma	Disconnected from school	Lives with parents	Child of migrants	Mother: Intermediate occupation Father: Tradesperson	Somatic symptom disorder
Hawa	17.5	Middle school diploma	In school	Lives with parents	Child of migrants	Mother: Employee Father: Employee	Somatic symptom disorder
Leslie	16.5	Middle school diploma	In school	Lives with parents	Child of migrants	Mother: Manager Father: Retired	Somatic symptom disorder
Lucie	19	Middle school diploma	In school	Parents separated, shared custody	0	Father: manager Mother: employee	Somatic symptom disorder
Aurélie	14.5	Middle school diploma	In school	Lives with parents	0	Father: manager Mother: employee	Somatic symptom disorder
Marie-Claire	15	Middle school diploma	In school	Parents separated; lives with her mother	Child of migrants	Father: chauffeur Mother: employee	Functional neurological disorder
Pauline	17	Nothing	Disconnected from school	Lives with spouse	Child of migrants	Father: unknown Mother: unknown	Functional neurological disorder
Samira	16.5	Middle school diploma	Disconnected from school	Lives with parents	Child of migrants	Father: employee Mother: unemployed	Functional neurological disorders
Donovan	17	Middle school diploma	In school	Lives with parents	Child of migrants	Father: manager Mother: manager	Psychological factors affecting other medical conditions (migraines)
Mehdi	14	Nothing	In school	Lives with parents	Child of migrants	Mother: employee Father: unknown	Illness anxiety disorder
Valentin	18	Baccalaureate	Disconnected from school	Lives with parents	0	–Mother: Intermediate occupation –Father: employee	Somatic symptom disorder
Myrtille	18	Middle school diploma	Disconnected from school	Lives with parents	0	–Mother: shopkeeper –Father: worker	Psychological factors affecting other medical conditions (rheumatoid arthritis)
Jade	18	French baccalaureate	In school	Parents separated	0	Father: manager Mother: intermediate occupation	Illness anxiety disorder
Sarah	20	Middle school diploma	Disconnected from school	Parents separated	Child of migrants	Mother: job hunting Father: manager	Factitious disorder
Marie-Lou	18	French baccalaureate	In school	Parents separated	0	–Mother: shopkeeper –Father: shopkeeper	Psychological factors affecting other medical conditions (Ehler Danlos)
Marie	14	Nothing	In school	Lives with paternal grandparents	0	–PGM: employee –PGF: intermediate occupation	Somatic symptom disorder
Maryam	15	Middle school diploma	In school	Lives with parents	Child of migrant	Father: manager Mother: employee	Functional neurological disorder
Marthe	21	Baccalaureate	Post-secondary studies	Lives with parents + maternal grandmother	Child of migrant	–Father: manager –Mother: employee	Somatic symptom disorder
Andréa	16	Middle school diploma	Disconnected from school	Lives with parents	0	Father: employee Mother: intermediate occupation intermediary	Somatic symptom disorder
Maxime	17	Middle school diploma	Disconnected from school	Lives with parents	0	Father: shopkeeper Mother: shopkeeper	Somatic symptom disorder
Manon	19	Baccalaureate	In school	Lives with her parents	Child of migrants (4th gen)	Father: manager Mother: employee	Somatic symptom disorder
Esther	17	Middle school diploma	In school	Lives with paternal grandmother	Migrant	PGM = employee	Functional neurological disorder
Marine	17	French baccalaureate	In school	Lives with parents	0	Mother: employee Father: employee	Functional neurological disorder
Nassim	15	Middle school diploma	In school	Lives with maternal grandmother	Child of migrants	Father: worker Mother: employee	Somatic symptom disorder
Yasmine	18	Middle school diploma	In school	Lives with a friend	Child of migrants	Mother: employee Father: employee	Functional neurological disorder

*First names anonymized

## Abbreviations

DSM: Diagnostic and statistical manual
SSD: Somatic symptom and related disorders
WHO ICD: World Health Organization International Classification of Diseases
BDD: Bodily distress disorders
FD: Functional disorders
COREQ: Consolidated criteria for Reporting Qualitative research
DCPR: Diagnostic criteria in psychosomatic research
AP-HP: Assistance Publique–Hôpitaux de Paris

## Footnotes

1 CERAPHP Center – IRB #00,011,928).

2 The cultural theories corresponding to etiological theories about disease — the meaning we give to what happens to us. This search for an individual meaning calls upon collective cultural representations.
